# Creutzfeldt–Jakob Disease: Spectrum of Symptoms, Clinical Progress and Diagnostics—Report of Five Cases

**DOI:** 10.3390/neurosci7020035

**Published:** 2026-03-10

**Authors:** Klaudia Rojewska, Natalia Dynowska, Iwona Rotter, Małgorzata Niekrasz

**Affiliations:** 1 Department of Neurology with Stroke Unit, Independent Public Voivodeship Combined Hospital in Szczecin, ul. Arkońska 4, 71-455 Szczecin, Poland; natdynowska@spwsz.szczecin.pl (N.D.);; 2 Department of Medical Rehabilitation and Clinical Physiotherapy, Pomeranian Medical University in Szczecin, ul. Żołnierska 54, 71-210 Szczecin, Poland

**Keywords:** Creutzfeldt–Jakob disease, protein 14-3-3, RT-QuIC, cortical ribbon sign, rapidly progressive dementia

## Abstract

Creutzfeldt–Jakob disease (CJD) is a rare, fatal prion disease of the central nervous system that develops due to the conversion of the normal cellular protein PrPc to the abnormal PrPSc molecule. The first clinical cases were described in the 1920s. The aim of this paper is to present the clinical progress of the disease and the diagnostic process, including some of the most common diagnostic traps. The paper highlights a range of symptoms that should serve as a potential warning signal for clinicians—not just neurologists—indicating the need to evaluate the patient more thoroughly.

## 1. Introduction

Creutzfeldt–Jakob disease (CJD) is a rare, fatal prion disease of the central nervous system that develops due to the conversion of the normal cellular protein PrPc to the abnormal PrPSc molecule. The first clinical cases were described in the 1920s [1].

The most recent outbreak of Creutzfeldt–Jakob disease reportedly took place between 1996 and 2003, with the highest number of cases recorded at that time in the UK and France. In a study collecting data from 1993 to 2020, a total of 27,872 cases of prion diseases were recorded across 34 countries, including 24,623 cases of sCJD, 485 cases of iCJD, and 232 cases of vCJD. Israel and Slovakia report a much higher percentage of genetic prion disease, accounting for over 60% of diagnosed cases [2] (Table 1). 

Over the past 27 years, the annual number of PrD cases and mortalities has shown an increasing trend. Currently, the incidence of the disease is estimated at 1–2 cases per million people. This may be related to the increased awareness among medical personnel of the clinical course of the disease, as well as the higher availability of diagnostic testing. Despite the increased number of registered cases, the etiology of prion diseases remains largely unexplored.

Given the heterogeneity of the clinical presentation, the differential diagnosis should include a number of diseases, primarily those with an acute onset, such as encephalitis or meningitis, autoimmune encephalitis, paraneoplastic syndrome, ischemic stroke, neurodegenerative disorders (including Parkinson’s disease, PSP, DLB), psychiatric disorders (including schizophrenia, bipolar disorder), and vitamin deficiencies [5,6] (Table 2).

In all the cases presented below, neurodegenerative, autoimmune, and inflammatory disorders were excluded.

The following article presents five clinical cases of patients diagnosed in the Neurology Department of SPWSZ in Szczecin over the course of a year and a half.

## 2. Case 1

A 65-year-old man with coronary artery disease, a history of coronary stenting, and no prior history of psychiatric illnesses, was first consulted by a neurologist in the Emergency Department on 8 January 2024. His reason for reporting to the hospital was a slowly increasing deterioration of speech fluency over the past month. Neurological examination confirmed impaired speech with predominant motor and amnestic aphasia—he was able to communicate in single words, but was unable to form extensive and complex statements. Comprehension and the ability to follow compounded commands were preserved. No paresis was observed, and muscle tone was assessed as normal. There was no indication of ataxia, and gait was normal without assistance.

A CT scan of the head was performed, revealing no pathological findings. An ischemic stroke with ensuing mild motor aphasia was suggested as the most likely diagnosis, and a referral for an outpatient brain MRI was issued. Patient was discharged from the ER with standard recommendations for a stroke patient.

On 26 January 2024, the patient was brought back to the Emergency Department by his family due to general weakness, gradual progression of speech impairment, and additional complaints of an increasing balance disorder, manifesting as unsteadiness and staggering, making unassisted walking impossible. Patient was often confused regarding time and place. The above symptoms had been intensifying for at least two months.

Upon admission, he was in a relatively good general condition, conscious, but presenting with occasional erratic behavior and auto- and allopsychic disorientation. He failed to communicate logically or follow instructions. His speech was dominated by verbal perseverations and echolalia. Muscle hypertonia was present in all four limbs, though more pronounced in the upper limbs. Patient was admitted to the Neurology Department.

For diagnostic purposes, a lumbar puncture was performed. First, any inflammatory changes in the CNS were ruled out; cytology and basic biochemistry results were normal. Autoimmune spectrum disorders were also ruled out with a screening panel for typical encephalitis and paraneoplastic syndrome antibodies, which yielded negative results.

**Figure 1:**
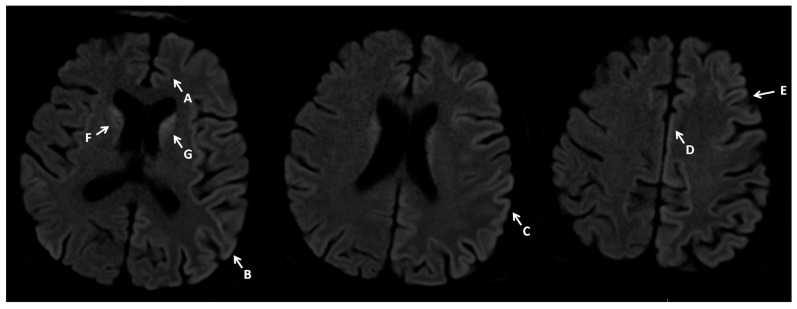
Contrast MRI imaging 7 February 2024. Imaging performed on a 1.5T scanner. Brain MRI shows increased signals on DWI diffusion images in the cerebral cortex—cortical ribbon sign (A–E) and caudate nuclei (F,G), with these changes being more pronounced in the left hemisphere.

A brain MRI with contrast was ordered next. The initial radiological report revealed small vessel disease, but no new vasculogenic lesions, focal lesions, or CNS inflammation were detected (Figure 1).

**Figure 2:**
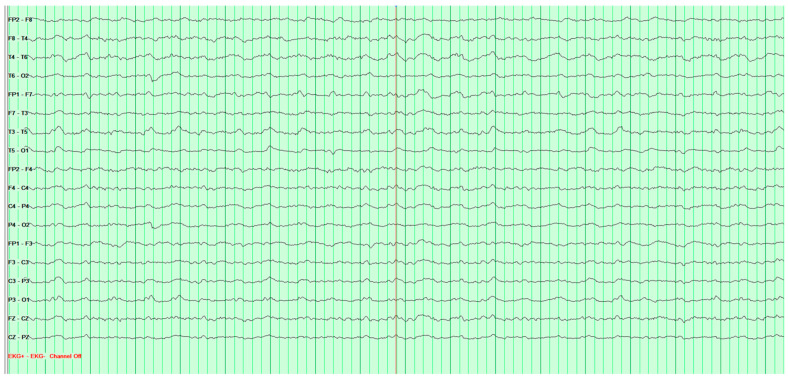
First EEG examination of a 65-year-old male patient, performed on 1 February 2024. Against the background of low-voltage basic activity visible alpha rhythm of 9–10 Hz, sequences of polymorphic slow waves at 2–3–4 Hz with amplitudes up to 170 μV are recorded over the left cerebral hemisphere. These waves show a tendency toward paroxysmal occurrence and generalization to other leads. No epileptiform discharges (Fs−). The study was performed without hyperventilation (Hv).

Throughout the hospitalization, altogether four EEG tests were performed. The first showed significant pathological changes in the leads above the left cerebral hemisphere, but gave no grounds for a definitive diagnosis (Figure 2).

Given the inconclusive character of the above results, a lumbar puncture was repeated—a test for the presence of 14-3-3 protein in the cerebrospinal fluid (CSF) was performed, yielding a strongly positive result.

**Figure 3:**
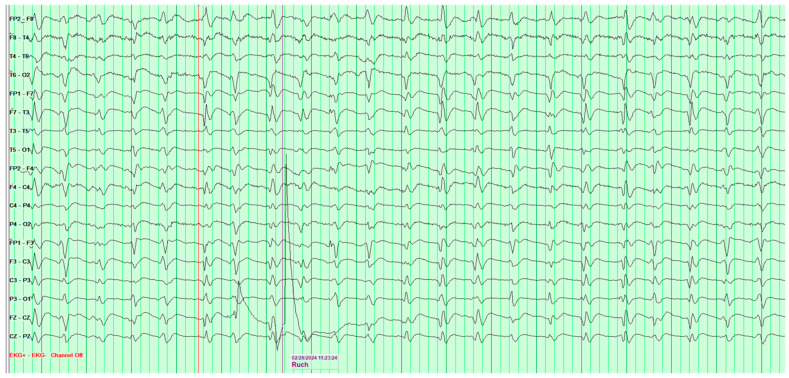
Fourth EEG examination of a 65-year-old male patient, performed on 28 February 2024. Against a background of low-voltage, fast basic activity, periodic generalized discharges of triphasic sharp waves with amplitudes up to 200 μV are recorded, recurring at 1l-s intervals. No epileptiform discharges (Fs−). The study was performed without photic stimulation (Rz) and without hyperventilation (Hv). EEG recording characteristic for Creutzfeldt-Jakob disease.

The following EEG tests showed a steady progression of pathological changes until a recording typical of Creutzfeldt–Jakob disease was documented (Figure 3).

Given the CSF test results, the progression of EEG changes, and the clinical picture, the MRI report was revised. Pathologies typical of Creutzfeldt–Jakob disease were retrospectively identified.

Over the next four weeks of hospitalization, the patient’s neurological condition gradually but undeniably deteriorated. Patient’s behavior at times suggested an anxiety disorder and visual hallucinations, yet he remained unable to logically communicate his ailments. He was consulted by a psychiatrist; any attempts to initiate antipsychotic treatment were unsuccessful. He remained confined to the bed; extrapyramidal rigidity was gradually progressing predominantly in the right limbs. Deliberate reflexes were observed. Myoclonus was noted to affect mostly the right limbs, as well as the intercostal and facial muscles. Speech disorder worsened, leading to complete akinetic mutism. Due to increasing dysphagia, he required the insertion of a gastric tube. 

According to the met criteria, a diagnosis of probable Creutzfeldt–Jakob disease was decided on. Treatment was continued in the Palliative Care Unit.

Despite ongoing medical care—neurological, psychiatric, and rehabilitation efforts—continued degeneration of the nervous system was observed, and the patient died four months after the start of hospitalization.

## 3. Case 2

A 50-year-old woman with no history of chronic illness, no essential medication, and no family history of psychiatric illness was admitted to the Neurology Department due to behavioral changes—irrational, anxious reactions to everyday situations, periodic disturbances in allopsychic orientation, short-term memory impairment, and chronic insomnia. These symptoms primarily concerned the patient’s family; subjectively, she did not report any ailments and did not feel the need to contact a physician.

She had no previous employment history and was responsible for providing childcare for her grandchildren and managing the household. In the two weeks preceding hospitalization, the patient required constant home care due to her progressive lack of independence. She required supervision to perform basic tasks at home, was often confused, and, according to her daughters, regularly uttered persecutory delusions. An episodic occurrence of Reichardt’s sign despite not consuming alcohol was also reported.

On admission day, neurological examination revealed mild spastic paresis of the left upper limb. Cerebellar ataxia was found in the lower limbs, predominantly on the left side. Gait was independent but unstable.

Significant emotional lability was observed—patient expressed suicidal thoughts and continued to express delusions. However, a sense of anosognosia and a lack of awareness regarding reasons for her hospitalization persisted. She alternated between confused, agitated wandering around the ward and a completely immobile state, with catatonic features—sitting in the dark, frozen mid-gesture. Severe insomnia was observed overnight. Speech was fluent yet digressive, with all unprovoked statements dominated by childhood memories and focusing primarily on the physical abuse she had experienced several years earlier.

Patient underwent a psychiatric consultation due to the initial suspicion of developing psychosis—the proposed treatment did not alleviate any of the described symptoms.

As in the first case, the CSF diagnostics included an assessment of basic cytology, biochemistry, and the presence of antibodies typical of encephalitis and paraneoplastic syndrome—the results were within normal range. However, the result for the presence of 14-3-3 protein in the CSF was determined to be very strongly positive.

**Figure 4:**
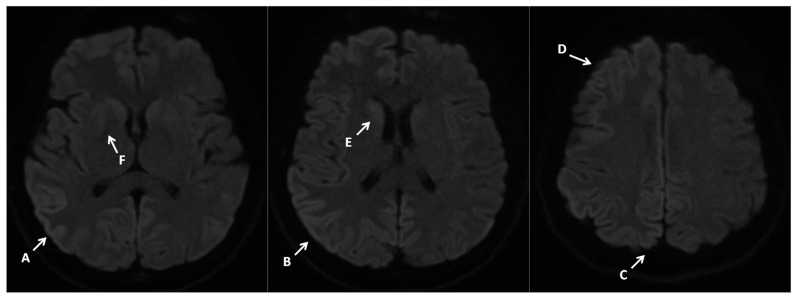
Contrast MRI imaging 5 April 2024. Imaging performed on a 1.5T scanner. Brain MRI shows abnormal imaging with increased signals on DWI imaging of the right cerebral cortex-cortical ribbon sign (A–D). Similar changes are also seen in the right caudate nucleus (E) and putamen (F) and medially in the left parietal lobe.

The MRI with contrast results revealed changes characteristic of Creutzfeldt–Jakob disease (Figure 4).

**Figure 5:**
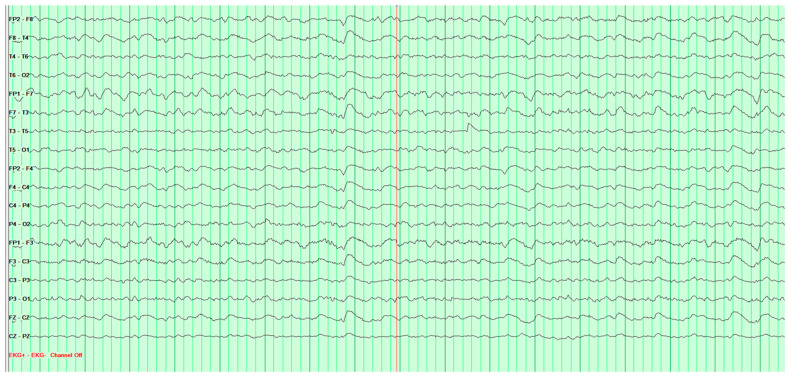
First EEG examination of 50-year-old female patient, performed on 14 March 2024. The basic activity is sporadically visible-fairly regular alpha rhythm at 9–10 Hz mixed with faster activity, with amplitudes up to 40 μV. Against this background, paroxysmal sequences of slow waves at 1.5–1.9 Hz, as well as abortive sharp wave-slow wave complexes at 1.7 Hz with amplitudes up to 220 μV, are recorded in all leads, with left-sided lateralization. No epileptiform discharges (Fs−). The study was performed without hyperventilation (Hv).

Throughout the hospitalization, altogether five EEG tests were performed. The first showed persistent slow-wave pathological activity predominating over the left hemisphere, but gave no grounds for a definitive diagnosis (Figure 5).

**Figure 6:**
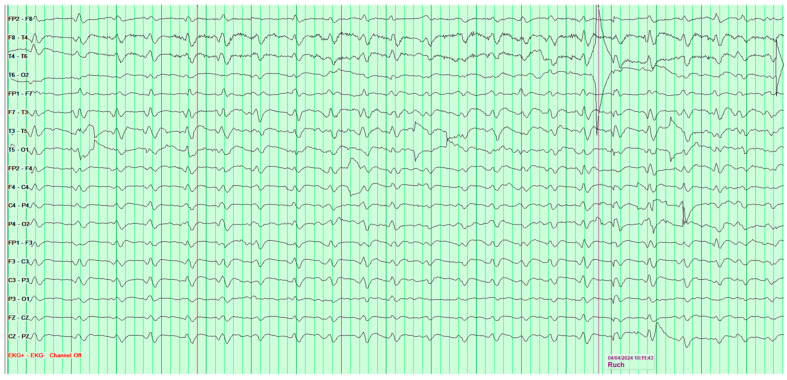
Fifth EEG examination of 50-year-old female patient, performed on 4 April 2024. Against a background of low-amplitude, predominantly fast basic activity, cyclic discharges of triphasic sharp waves with an amplitude of up to 190 μV are recorded every approximately 1 s. (Fs−). The recording was performed without photic stimulation or hyperventilation. EEG recording characteristic for Creutzfeldt-Jakob disease.

EEG test performed 4 weeks after the first one showed a recording typical of Creutzfeldt–Jakob disease (Figure 6).

According to the met criteria, a diagnosis of probable Creutzfeldt–Jakob disease was decided on. Over the next two weeks, very rapid neurological deterioration was observed: significant delay in responding to questions, bradyphrenia, adynamia, apraxia, and worsening deficits in episodic, short-term, and autobiographical memory. Psychological support and cognitive exercises did not improve cognitive function.

In the third week of hospitalization, the patient remained bedridden, requiring comprehensive care and nursing, failing to establish logical communication—spastic quadriparesis predominantly in the left limbs was present. Generalized myoclonus was observed as well as akinetic mutism. Due to dysphagia, she required the insertion of a gastric tube.

The patient died on the 36th day of hospitalization in the Neurology Department (Table 3).

Due to the wishes of the patient’s family, the post-mortem examination was waived in the above-mentioned case.

## 4. Case 3

A 72-year-old patient with hypertension and type 2 diabetes was admitted to the Neurology Department to further the diagnosis of significant muscle hypertonia affecting all four limbs, which had been progressing for seven months, as well as gait impairment and visual hallucinations. A year prior to the admission, extrapyramidal syndrome had been diagnosed and treatment implemented, without improvement. In order to verify the pharmacotherapy and eliminate possible drug complications, the previous medications were discontinued upon admission.

Patient was a farmer engaged in agriculture and livestock breeding. There was no determined family history of psychiatric illness.

She was conscious and able to answer simple and direct questions; further neurological examination revealed abnormalities typical of extrapyramidal syndrome: muscle rigidity present in all four limbs, predominantly on the left, with a spastic contracture of the left hand; tremor present in both upper limbs and the mandible; apraxia; bradykinesia; slowed speech; hypomimia. Despite the absence of objective paresis, the patient used a wheelchair.

A lumbar puncture was performed, and the CSF diagnostics included general tests, which revealed normal cytology and a slightly elevated glucose level. The presence of antibodies typical of encephalitis and paraneoplastic syndrome was ruled out. Neuroborreliosis was ruled out. Test for the presence of 14-3-3 protein yielded positive results.

**Figure 7:**
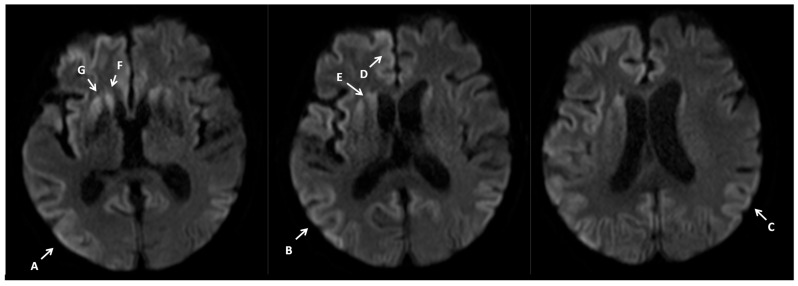
Contrast MRI imaging 10 June 2024. Imaging performed on a 3T scanner. Brain MRI shows diffuse increased signal on diffusion-weighted imaging (DWI) within the cerebral cortsex-cortical ribbon sign (A–D), caudate nuclei (E,F), and putamen (G), with changes more pronounced on the right side. Imaging burdened with movement artifacts.

A contrast MRI of the brain was performed, revealing pathologies typical of Creutzfeldt–Jakob disease (Figure 7).

**Figure 8:**
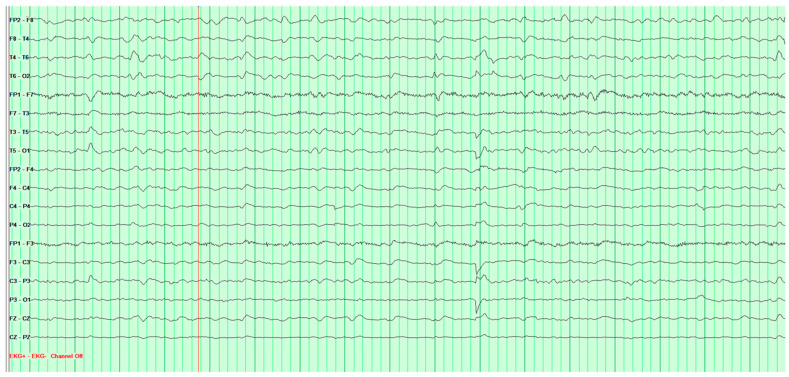
First EEG examination of 72-year-old female patient performed on 6 June 2024. The recording is spatially non-differentiated. The basic activity in the parietal-occipital regions is markedly slowed, consisting of theta waves at 5 Hz with amplitudes up to 40 μV. In the frontal regions, theta waves at 5–7 Hz with amplitudes up to 30 μV are recorded. No alpha rhythm is observed. Against this background, single and grouped sharp waves of higher amplitude than the background are visible in all regions, occasionally with a tendency toward generalization. Hyperventilation and photic stimulation were not performed due to lack of cooperation. Sings of suspected Creutzfeldt-Jkcob disease.

**Figure 9:**
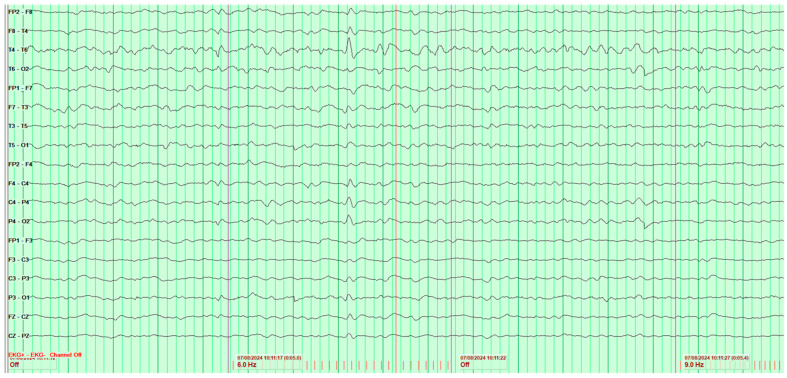
Fourth EEG examination of a 72-year-old female patient performed on 8 July 2024. The recording is spatially non-differentiated. The basic activity in the parietal-occipital regions is markedly slowed, consisting of theta waves at 5–6 Hz with amplitudes up to 40 μV. In the frontal regions, theta waves at 5–7 Hz with amplitudes up to 30 μV are recorded. No alpha rhythm is observed. Against the background, single and grouped triphasic sharp waves of higher amplitude than the background are seen in the right centrotemporal region, occasionally with a tendency toward generalization. Hyperventilation and photic stimulation were not performed due to lack of cooperation. Sings of suspected Creutzfeldt-Jkcob disease.

Throughout the hospitalization, altogether four EEG tests were performed. The first showed signs of suspected Creutzfeldt–Jakob disease (Figure 8 and Figure 9).

During the subsequent days of hospitalization, due to increasing apraxia and despite rehabilitation, the patient became bedridden and completely dependent on others. Initially observed tremors slightly worsened, and the generalized rigidity increased.

She received care and support from a psychologist, who described her interactions with the patient as fluctuating, periodically superficial—she was able to follow simple commands, but was illogical; logorrhea, with a tendency to express incoherent and confused thoughts, dominated her speech. Her perception of the environment was fragmented with limited criticism, and she occasionally presented delusional interpretations of the surrounding reality. At times, the patient appeared to be experiencing visual hallucinations.

According to the met criteria, a diagnosis of probable Creutzfeldt–Jakob disease was decided on. The patient was hospitalized in the Neurology Department for 32 days, during which her neurological condition slightly deteriorated, without any drastic and rapid changes. She was discharged into the care of her family in a stable general condition. According to the family, in the following weeks, logorrhea slowly morphed into akinetic mutism. No accompanying myoclonus was observed. She died at home on 15 October 2024, about two years after the first extrapyramidal symptoms were noted.

## 5. Case 4

A 67-year-old woman, a retired teacher with hypothyroidism, was admitted to the Neurology Department on 6 February 2025, for further evaluation of short-term memory loss and progressive disorientation that she had been experiencing for a month and a half. Additionally, she reported rapidly progressing apraxia, agraphia, motor aphasia, and periodic visual hallucinations. There was no family history of mental illness.

A neurological examination on the day of admission revealed that the patient was able to follow simple commands, her speech was slowed and scanning, her muscle tone was decreased in all four limbs, and a cerebellar tremor of the upper limbs was present, predominantly on the left side. Additionally, there was a tremor in the mandible. Involuntary chorea of the upper limbs was noted, especially prominent during attempts at walking. Gait was unstable and required assistance. During the whole examination, the patient was anxious but fully aware of her ailments.

A lumbar puncture was performed, ruling out meningitis, encephalitis, neuroborreliosis, autoimmune disorders, and paraneoplastic syndrome. Test for the presence of 14-3-3 in CSF protein yielded positive results.

**Figure 10:**
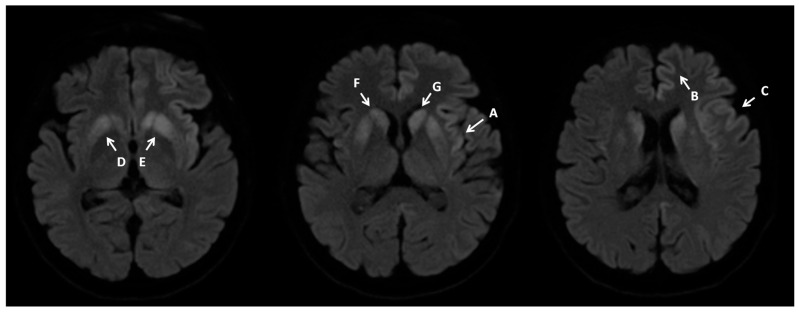
Contrast MRI imaging 12 February 2025. Imaging performed on a 3T scanner. Brain MRI shows increased signal on diffusion-weighted imaging (DWI) in the cortical gyri of the left hemisphere more intense in the area of the insular cortex (A)-cortical ribbon sign (B,C). Furthermore relatively symmetrical increased signals are visible in the lenticular nuclei (D,E) and the head of the caudate nucleus (F,G).

A contrast MRI of the brain was performed, revealing pathologies typical of Creutzfeldt–Jakob disease (Figure 10).

**Figure 11:**
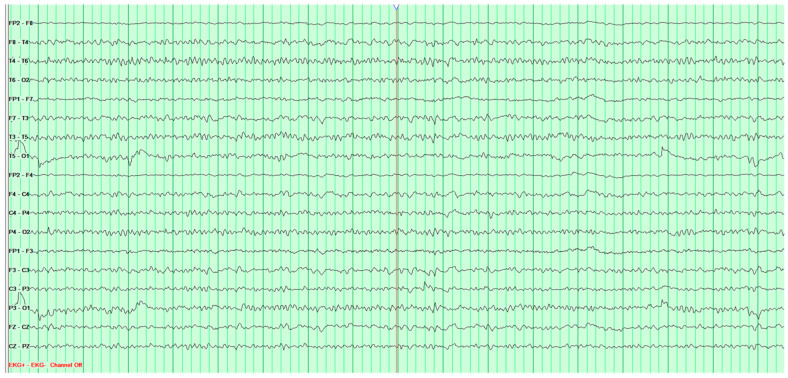
First EEG examination of 67-year-old female patient performed on 10 February 2025. The examination was performed during wakefulness. The recording is spatially differentiated. The basic activity consist of alpha waves at 9 Hz with amplitude up to 80 μV. The alpha rhythm well expressed. Against the background, in all derivations−with variable lateral predominance-paroxysmal discharges of spikes, sharp waves and groups of slow waves at 2.3–2.6 Hz with amplitude up to 190 μV are recorded. Hyperventilation and photic stimulation had no effect on recording.

**Figure 12:**
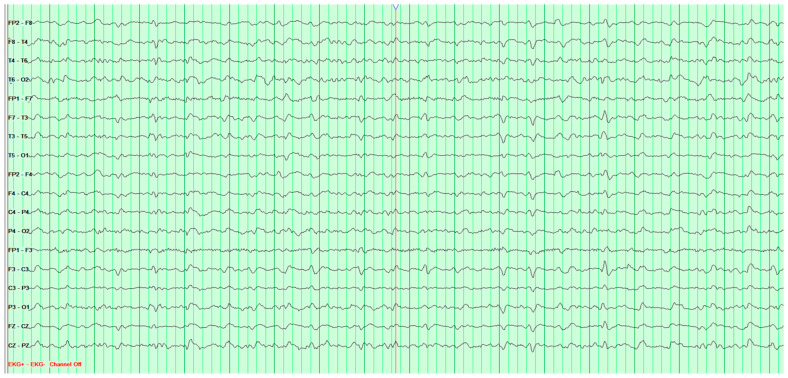
Second EEG examination of 67-year-old female patient performed on 17 February 2025. The examination was performed during wakefulness. The recording is spatially differentiated. The basic activity consist of alpha waves at 8–9 Hz with amplitude up to 40 μV. The alpha rhythm is well expressed. Against the background, in all derivations at irregular intervals, paroxysmal discharges of polymorphic slow waves (2–3 Hz), sharp waves, spikes and triphasic sharp waves with amplitude up to 160 μV are observed. Photic Sighs of suspected Creutzfeldt-Jkcob disease.

Two EEG tests were performed, and the first one showed generalized seizure activity that required further monitoring (Figure 11). A week later, the second test showed signs of suspected Creutzfeldt–Jakob disease (Figure 12).

During hospitalization, haloperidol was initiated, resulting in a reduction of chorea. Anti-anxiety treatment was also necessary. During patient’s eleven-day stay in the Neurology Department, substantial deterioration of her condition was observed. Her speech disorder worsened, with motor aphasia predominating; patient was unable to form complex, long sentences. Vocalization was significantly delayed. A decline in visual and cognitive functions was described, with a global cognitive performance index of 47% out of 100%. Ataxia made most intentional activities impossible to accomplish without assistance. Patient was auto- and allopsychologically oriented throughout the whole hospitalization, aware of her symptoms and prognosis—she remained under constant psychological care.

According to the met criteria, a diagnosis of probable Creutzfeldt–Jakob disease was decided on. After completing the diagnostic process, the patient was discharged home into the care of her family. Three days after discharge, the patient’s husband reported a significant deterioration in her neurological condition: she became unresponsive to external stimuli, presented with akinetic mutism, and required a nasogastric tube due to dysphagia. The patient was admitted to a hospice, where she died after three weeks of palliative care.

## 6. Case 5

A 72-year-old man, a retired warehouse worker, was admitted to the Neurology Department to further the diagnosis of a speech disorder.

The patient had an extensive history of vascular diseases: hypertension, aneurysms of the left popliteal artery and the right common iliac artery—both with stents; right kidney infarction; one episode of upper gastrointestinal bleeding. There was no known history of psychiatric illness in the family.

The patient reported difficulty speaking and finding the correct words to express himself; he began noticing these symptoms within the week before hospitalization. He also complained of short-term memory loss and vision impairment in the form of blurring. The patient’s family assessed all of the above symptoms, especially speech impairment, as progressive.

Neurological examination on the day of admission revealed the patient to be conscious and logical, with slowed, scanning speech and signs of sensory aphasia; echolalia and perseverations were present. Beginnings of agraphia and acalculia were observed. Ataxia in the right limbs was noted. Gait remained unassisted. During the psychological assessment, significant short-term memory loss was described.

A lumbar puncture was performed, ruling out meningitis, encephalitis, neuroborreliosis, autoimmune disorders, and paraneoplastic syndrome. Further diagnostic workup included sending the cerebrospinal fluid for 14-3-3 protein testing and an RT-QuIC test. The 14-3-3 protein test was negative, while the RT-QuIC test, the gold standard for CJD diagnosis, was unequivocally positive.

**Figure 13:**
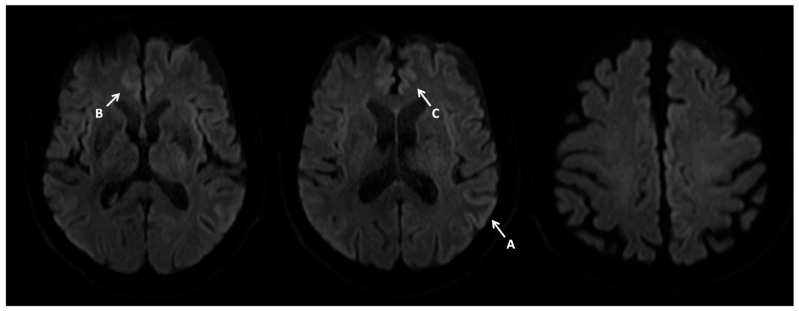
Contrast MRI imaging 23 May 2025. Imaging performed on a 3T scanner. Brain MRI shows moderate diffusion restriction in the cortical layer of the lateral part of the left temporal lobe (A), overlapping a small portion of the parietal cortex, with no detectable changes on Flair and T2 images. Slight cortical signal enhancement in the medial frontal gyri on DWI images (B,C), without significant signal reduction on ADC maps.

A contrast-enhanced MRI of the brain was performed. The initial radiological report revealed several lesions representing secondary vasogenic changes in small vessels (grade 1 according to the Fazekas scale). No changes typical of CJD were described. However, given the remaining test results and the patient’s clinical condition, the report was retrospectively revised. The image was deemed nonspecific; yet, considering the clinical data, the initial changes in the course of Creutzfeldt–Jakob disease should be the primary consideration (Figure 13).

**Figure 14:**
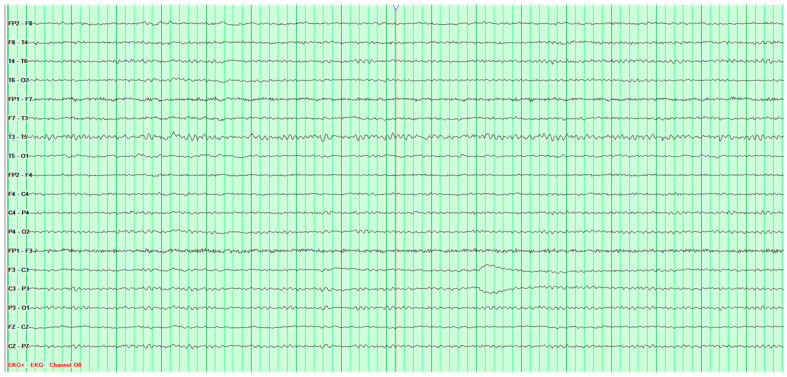
First EEG examination of 72-year-old male patient performed on 21 May 2025. The examination was performed during wakefulness. The recording contains ocular artifacts and is spatially differentiated. The basic activity consist of alpha waves at 8–9 Hz with an amplitude up to 70 μV. The alpha rhythm is well expressed. Against the background, scattered sharp waves with amplitude up to 100 μV are recorded, predominantly in posterior leads. No focal pathology or epileptiform activity is observed. Hyperventilation and photic stimulation had no effect on the recording.

**Figure 15:**
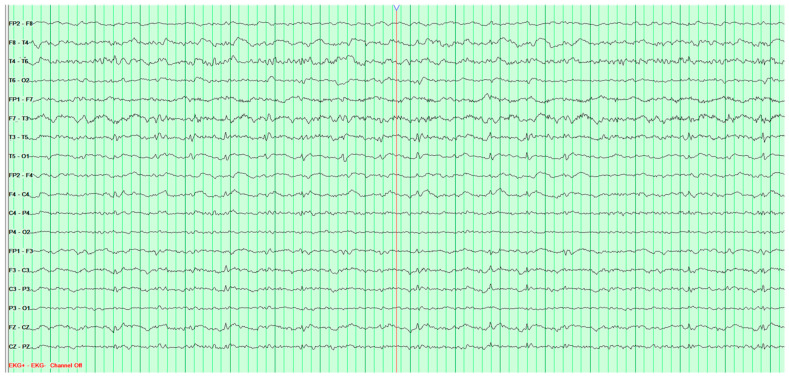
Second EEG examination of 72-year-old male patient performed on 9 June 2025. The examination was performed during wakefulness. The basic activity consist of alpha waves at 8–9 Hz with amplitude up to 40 μV, intermixed with low-amplitude fast activity. The alpha rhythm is absent. Against the background in all derivations with predominance over he left cerebral hemisphere groups of slow waves at 3–4 Hz and spike-slow wave complexes at 4 Hz with amplitude up to 60 μV are recorded. Hyperventilation and photic stimulation had no effect on the recording.

Two EEG tests were performed, neither of which showed definitive pathologies typical of Creutzfeldt–Jakob disease (Figure 14 and Figure 15).

According to the met criteria, a diagnosis of probable Creutzfeldt–Jakob disease was decided on. During his stay in the Neurology Department, the patient received speech therapy and psychological care. Despite these efforts, there was no improvement in his cognitive function.

He was discharged home into the care of his family after 11 days of hospitalization. According to his wife, his speech impairment worsened over time, leading to akinetic mutism. He was unable to walk independently due to general confusion rather than a mobility impairment, requiring the use of a wheelchair. Before the occurrence of dysphagia, the patient refused to eat despite having the ability to. The first episode of choking resulted in aspiration pneumonia, and he was admitted to the Internal Medicine Department, where he died on 15 July 2025.

## 7. Conclusions

Given the examples presented above, it is essential to consider CJD in the differential diagnosis of patients with rapidly progressing dementia. It poses significant diagnostic challenges, as sometimes the psychiatric symptoms may mask the underlying neurological condition. In patients with drug-resistant psychotic symptoms, a lumbar puncture should be considered before further escalation of pharmacotherapy.

Each of the presented patients initially manifested symptoms of rapidly progressing dementia and apraxia. Anxiety disorder and visual hallucinations were also common denominators between cases, with paranoid delusions occurring rarely. Cerebellar signs were more common than pyramidal or extrapyramidal syndrome. Myoclonia and akinetic mutism remain the most typical neurological symptoms to pay attention to (Table 4 and Table 5).

The diagnostic workup should first include a lumbar puncture and a comprehensive range of cerebrospinal fluid tests. Inflammatory, autoimmune, and paraneoplastic diseases should be ruled out. If negative results are obtained, further tests should be performed to evaluate the presence of 14-3-3 protein and perform the RT-QuIC test [7,8,9]. The latter is considered the gold standard for diagnosing prion diseases, and as presented in the fifth case, it can be the deciding factor when establishing a diagnosis. RT-QuIC is considered more specific than the 14-3-3 protein, and positive results can be obtained even when the presence of the protein is ruled out [10]. Despite the RT-QuIC test being unavailable in Poland, we ensured cooperation with a laboratory in Prague, Czech Republic, due to the high diagnostic value of the test.

An EEG is an important supplementary test that requires repetition, as the pathologies typical of CJD can become evident in the later stages of the disease in accordance with the deterioration of the patient’s general condition. In some cases, the results may not be conclusive.

A CT scan of the head is non-diagnostic in this instance; an MRI with contrast is recommended, preferably on a 3T machine if available. Collaboration between a radiologist and a neurologist can prove pivotal when reviewing radiological findings. Correlation between the clinical presentation and even the slightest changes found on imaging can facilitate an accurate interpretation of the test.

In patients diagnosed with prion disease, a multidisciplinary approach—including psychiatric, psychological, neurological, and rehabilitation efforts, as well as speech therapy—can help with managing symptoms and improving the patient’s comfort during end-of-life care. Equally important is providing support for the patient’s family.

Given the rapid course of the disease and the potential spread of the infection, every case should be reported to the epidemiological control centers specific to each country. Consistent reporting can prevent further outbreaks and can lead to collective data analysis to identify common causes or triggers. In the coming years, this may become the basis for identifying methods to prevent the disease. Testing cattle for prion diseases is also crucial, as it significantly minimizes the incidence of vCJD.

**Table 4:** Neurological symptoms [11].

	Primitive Reflexes	Apraxia	Akinetic Mutism	Myoclonia	Extrapyramidal Syndrome	Pyramidal Syndrome	Cerebellar Signs
M 65	YES	YES	YES	YES	YES	YES	NO
W 50	NO	YES	YES	YES	NO	YES	YES
W 72	YES	YES	NO	YES	YES	NO	YES
W 67	NO	YES	YES	YES	NO	NO	YES
M 73	NO	YES	YES	NO	NO	NO	YES

**Table 5:** Psychiatric symptoms [12].

	Insomnia	Suicidal Thoughts	Anxiety Disorders	Echolalia	Paranoid Delusions	Visual Hallucinations	Dementia
M 65	YES	NO	YES	YES	NO	YES	YES
W 50	YES	YES	YES	YES	YES	YES	YES
W 72	NO	YES	YES	NO	NO	YES	YES
W 67	NO	NO	YES	NO	NO	YES	YES
M 73	NO	NO	NO	YES	NO	NO	YES

## Figures and Tables

**Table 1:** Classification of CJD [3,4].

Classification of CJD	
1. Sporadic (Idiopathic)	Sporadic CJD (sCJD) accounts for approximately 80–90% of all diagnosed cases. It results from a spontaneous mutation of the prion protein gene.
2. Genetic (Familial)	Familial CJD (fCJD) represents about 12% of diagnosed cases and is associated with several possible mutations in the PRNP gene.
Other hereditary prion diseases include: GSS Gerstmann–Straussler–Scheinker Syndrome FFI Fatal Familial Insomnia	
3. Acquired	Acquired forms of CJD are linked to neurosurgical procedures (e.g., corneal or dura mater transplants) or consumption of prion-contaminated animal products.
The iatrogenic form (iCJD) has been documented in approximately 200 cases worldwide to date. Variant forms of CJD (including atypical or acquired variants such as variant CJD associated with bovine spongiform encephalopathy).	

**Table 2:** Clinical Diagnostic Criteria for the Sporadic Form of Creutzfeldt–Jakob Disease (sCJD) [5,6].

Clinical Diagnostic Criteria for the Sporadic Form of Creutzfeldt–Jakob Disease (sCJD)	
1. Rapidly progressive dementia	
2. Neurological symptoms: Myoclonia Visual or cerebellar disturbances Pyramidal or extrapyramidal signs Akinetic mutism	
3. Characteristic findings in at least one of the following investigations: Typical EEG pattern, or Typical MRI findings, or Elevated 14-3-3 protein concentration in cerebrospinal fluid, or Positive test RT-QuIC	
4. Disease duration less than 2 years.	
Possible CJD	Point 1 + Point 2 (at least two neurological symptoms)
Probable CJD	Criteria fulfilled: Point 1 + Point 2 (at least two neurological symptoms) + Point 3 (at least two positive investigations), or Point 1 + Point 2 (at least two neurological symptoms) + Point 3 (one positive investigation) + Point 4
Definite CJD	Presence of characteristic histopathological changes and/or prion protein deposits confirmed by neuropathological examination.

**Table 3:** Fulminant course of the disease.

Progression of Symptoms	Date
Independent walk	07.03
Walking with support against the wall	14.03
Requires diapering	18.03
First difficulties with eating—“I don’t know how to use bread”	20.03
Walking impossible. Bedridden patient	22.03
Does not eat independently, requires feeding	25.03
EEG recording typical of CJD	27.03
Dysphagia, nasogastric tube placement	28.03
A strongly positive result for 14-3-3 protein, myoclonus observed	03.04
Macrocytic anemia, elevated liver function parameters	04.04
Patient’s death	12.04

## Data Availability

No new data were created or analyzed in this study. Data sharing is not applicable to this article.

## Abbreviations

The following abbreviations are used in this manuscript:

CJD	Creutzfeldt–Jakob disease
EEG	Electroencephalography
MRI	Magnetic resonance imaging
